# Determinants of left ventricular mass in obesity; a cardiovascular magnetic resonance study

**DOI:** 10.1186/1532-429X-11-9

**Published:** 2009-04-24

**Authors:** Oliver J Rider, Jane M Francis, Mohammed K Ali, James Byrne, Kieran Clarke, Stefan Neubauer, Steffen E Petersen

**Affiliations:** 1 Oxford Centre for Clinical Magnetic Resonance Research, University of Oxford, Oxford, UK; 2 Department of Physiology, Anatomy and Genetics, University of Oxford, Oxford, UK; 3 Department of Upper Gastrointestinal Surgery, Southampton, UK

## Abstract

**Background:**

Obesity is linked to increased left ventricular mass, an independent predictor of mortality. As a result of this, understanding the determinants of left ventricular mass in the setting of obesity has both therapeutic and prognostic implications. Using cardiovascular magnetic resonance our goal was to elucidate the main predictors of left ventricular mass in severely obese subjects free of additional cardiovascular risk factors.

**Methods:**

38 obese (BMI 37.8 ± 6.9 kg/m^2^) and 16 normal weight controls subjects, (BMI 21.7 ± 1.8 kg/m^2^), all without cardiovascular risk factors, underwent cardiovascular magnetic resonance imaging to assess left ventricular mass, left ventricular volumes and visceral fat mass. Left ventricular mass was then compared to serum and anthropometric markers of obesity linked to left ventricular mass, i.e. height, age, blood pressure, total fat mass, visceral fat mass, lean mass, serum leptin and fasting insulin level.

**Results:**

As expected, obesity was associated with significantly increased left ventricular mass (126 ± 27 vs 90 ± 20 g; p < 0.001). Stepwise multiple regression analysis showed that over 75% of the cross sectional variation in left ventricular mass can be explained by lean body mass (β = 0.51, p < 0.001), LV stroke volume (β = 0.31 p = 0.001) and abdominal visceral fat mass (β = 0.20, p = 0.02), all of which showed highly significant independent associations with left ventricular mass (overall R^2 ^= 0.77).

**Conclusion:**

The left ventricular hypertrophic response to obesity in the absence of additional cardiovascular risk factors is mainly attributable to increases in lean body mass, LV stroke volume and visceral fat mass. In view of the well documented link between obesity, left ventricular hypertrophy and mortality, these findings have potentially important prognostic and therapeutic implications for primary and secondary prevention.

## Background

Left ventricular hypertrophy is one of the characteristic cardiac adaptations to obesity. [1-3] As there is now a growing body of literature that has demonstrated a strong relationship between left ventricular hypertrophy and all cause mortality, [4-6] and given the increasing prevalence of obesity, and the fact that obesity is associated with an increased risk of death [7], understanding the ways in which obesity modulates cardiovascular risk is of increasing clinical importance. Furthermore, identification of the determinants of left ventricular mass in obesity has potentially important implications for prognosis and therapeutic intervention aimed at primary and secondary prevention.

The mechanisms underlying left ventricular hypertrophy have been the focus of many investigations and large population based studies have demonstrated that multiple clinical parameters are associated with left ventricular mass, such as age, systolic blood pressure, body size, and both visceral and free fat mass. [8] However, most of these studies have relied on echocardiography, a difficult technique in obesity due to thoracic fat deposits limiting acoustic windows. In addition to this, the majority of previous studies have investigated subjects with the obesity related co-morbidities of hypertension, diabetes and hypercholesterolaemia, factors that are known to exert independent effects on the cardiovascular system.

The excess fat mass associated with obesity is known to increase metabolic demand and, thus, both cardiac output and total blood volume are elevated in obesity. These circulatory changes cause left ventricular geometric remodelling in the form of cavity dilatation, a structural change commonly seen in obesity, which is then thought to lead to a compensatory left ventricular hypertrophic response in response to increased wall stress. [1,9-11]

In addition to this, advances in the understanding of hormonal changes in obesity have highlighted several alternative mechanisms. Increased visceral and subcutaneous adiposity is known to cause higher levels of serum leptin, the hallmark of human obesity, and hyperinsulinaemia, both of which have been linked to ventricular hypertrophy in humans and in animal models. [12-15]

Or aim was to identify the determinants of left ventricular mass in a population of otherwise healthy obese subjects, free from identifiable cardiovascular risk factors and co-morbidity. In order to achieve this, we used cardiovascular magnetic resonance (CMR), which yields accurate and highly reproducible assessment of left ventricular mass, regardless of the amount of chest wall fat, [16] and related these measures to age, height, body surface area, visceral fat mass, total fat mass, lean body mass, leptin, insulin, C-reactive protein, end-diastolic volume, stroke-volume and ascending aortic size and distensibility, all of which have been proposed as independent predictors of left ventricular mass.[8,14,17]

## Methods

### Ethics and Study Cohort

The study was approved by the local ethics committee, and informed written consent was obtained from each patient.

In this study thirty eight healthy obese subjects (BMI > 30 kg/m^2^, 9 male, 29 female) and sixteen normal weight subjects (BMI 18.5 – 24.9 kg/m^2^5 male, 11 female) were included into the study. In order to prevent the confounder of ethnicity all recruited subjects were Caucasian. All subjects were screened for the presence of identifiable cardiac risk factors and excluded if they had a history of cardiovascular disease, hypertension, diabetes, current smoking, or use of cardiac medications. All subjects had a normal 12 lead electrocardiogram and were normotensive at the time of scanning (averaged over three supine measures taken within 10 minutes) with no historical evidence of hypertension. Subjects were excluded if they had either an elevated fasting glucose level (≥ 6.7 mmol) or a fasting total cholesterol level ≥ 6.5 mmol, a history of coronary artery disease, or of cardiac chest pain or valvular heart disease. All patients were excluded if they had clinical or historical evidence of obstructive sleep apnoea. All subjects were limited to three thirty minute sessions of sweat producing exercise per week, and excluded if physical activity exceeded this level.

### Blood tests

Fasting blood tests for glucose, cholesterol, leptin and insulin, were taken on the day of the scan. Patients were asked to be fasted for at least 8 hours. An estimate of insulin resistance was calculated using the HOMA-IR equation (fasting insulin (μU/ml) × fasting glucose (mmol/l)]/22.5)[18,19].

### Body Composition analysis

Bio-electrical impedance was used to determine total body fat mass, and lean body mass using Bodystat ^©^1500 analyser. The use of Bioimpednace analysis has become routine in clinical research investigating body composition analysis. Although not the gold standard for analysis of body composition it has been proved to have good correlation with DEXA assessments in multiple studies. [20-22] Bio-electrical impedance analysis measures the impedance or opposition to the flow of an electric current through the body fluids contained mainly in the lean and fat tissue. Impedance is low in lean tissue, where intracellular and extracellular fluid and electrolytes are primarily contained, but high in fat tissue. Impedance is thus proportional to body water volume. In practice, a small 400 uA current at 50 kHz, was passed between electrodes spanning the body to provide a measure of impedance. The Bodystat ^©^1500 then converted measured impedance to a corresponding estimate of TBW. Lean body mass is then calculated from this estimate using an assumed hydration fraction for lean tissue. Fat mass is calculated as the difference between body weight and lean body mass. For the calculation of the waist:hip ratio the average of three waist measurements was recorded at a) the level of the umbilicus, and b) the level of the greater trochanter of the femur.

### CMR

#### Cardiac and Aortic Imaging

All CMR scans for the assessment of left ventricular mass, volumes, ejection fraction and aortic distensibility were performed on a 1.5 Tesla MR system (Siemens Medical Solutions, Erlangen, Germany) as previously described.[23] All imaging was prospectively cardiac gated with a precordial four lead ECG and acquired during end expiratory breathold. Cardiac cine images were acquired using a steady state free precession (SSFP) sequence with an echo time (TE) of 1.5 ms, a repetition time (TR) of 3.0 ms, temporal resolution 47.84 ms and a flip angle of 60°. Following localisation images, an SSFP cine short axis stack of contiguous images was obtained with a slice thickness of 7 mm and an interslice gap of 3 mm, as previously described. [24-26] Ascending aortic distensibility was determined from cine images taken at the level of the pulmonary artery as previously described. [25] Indices of aortic function were assessed using a 25 frame retrogated SSFP cine sequence with the following parameters: TR 42 ms, TE 1.4 ms, FOVread 380 mm, in plane resolution 1.97 mm, slice thickness 7 mm.

#### Visceral fat mass

A single breathold, contiguous 5 slice, T1 weighted Turbo Spin Echo sequence centred around the vertebral body of L5 (turbo factor 5, echo time TE 12 ms, TR 200 ms, slice thickness 10 mm) was modified so that the sequence served to suppress predominantly the water signal[27]. Transverse slices were then manually contoured to provide a visceral fat volume.

### Data Analysis

Image analysis for left ventricular volumes and mass was performed using Siemens analytical software (ARGUS^©^) as described [24]. The short axis stack was analysed manually contouring the endocardial borders from base to apex at end-diastole and end-systole. The epicardial border was contoured at end-diastole to yield myocardial mass. Left ventricular mass (g) were calculated as the epicardial volume minus the endocardial volume multiplied by 1.05 (specific gravity of myocardium). Cardiac output (l/min) was calculated as left ventricular stroke volume (ml) × heart rate (bpm).

### Statistical Analysis

All statistical analysis was performed using SPSS statistical software (version 15.0; SPSS Inc., Chicago, Ill., USA). Data are expressed as means ± SD unless otherwise stated. All continuous variables were normally distributed. Differences between groups were assessed by Student's unpaired t test. Obese and normal weight groups were matched as groups, not as pairs. A probability value of p < 0.05 was considered significant and two-tailed p values were used for all statistics.

To assess the major determinants of left ventricular mass, a stepwise multiple linear regression model was performed. This multivariate model consisted of left ventricular mass as dependent variable and of independent variables that had a significant relation with left ventricular mass in the simple linear regression analysis.

## Results

### Baseline Group Characteristics

**Figure 1:**
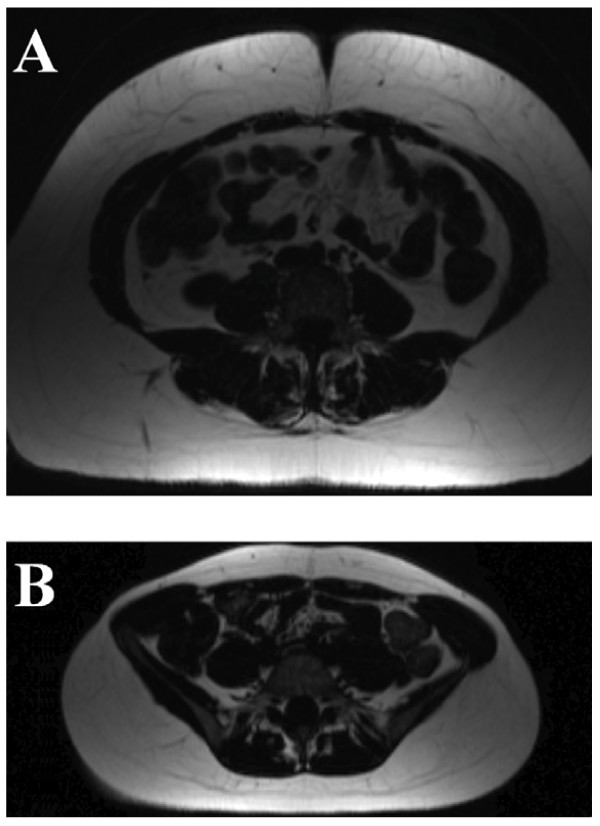
**Visceral Fat Mass Images from A) Obese (BMI 37.3 kg/m^2^) and B) Normal Weight Subject (BMI 19.5 kg/m^2^)**.

Normal controls and obese subjects were well matched for age, height, systolic blood pressure, diastolic blood pressure, fasting glucose and fasting total cholesterol, with no significant differences between obese and normal weight individuals. Waist hip ratio, total fat mass, visceral fat mass and lean mass were all higher in the obese group (Table 1 and 2, Figure 1). Serum leptin was over 9 times higher in the obese group. Fasting insulin and HOMA-IR were also higher in the obese cohort with the average still in the insulin sensitive range. C- reactive protein levels were increased more than 10-fold in the obese group (Tables 1 and 2).

**Table 1:** Anthropometric results for the study population

	Normal Weight Subjects N = 16	Obese Subjects N = 38	p value
Body Mass Index (kg/m^2^)	21.7 ± 1.8	37.8 ± 6.9	<0.001
Weight (kg)	61 ± 9	105 ± 18	<0.001
Percentage Excess Weight (%)	______	51 ± 28	
Systolic Blood Pressure (mmHg)	114 ± 9	117 ± 11	0.34
Diastolic Blood Pressure (mmHg)	72 ± 8	75 ± 8	0.21
Waist Hip Ratio	0.81 ± 0.08	0.92 ± 0.08	<0.001
Total Fat Mass (kg)	19 ± 10	47 ± 16	<0.001
Visceral Fat Mass (dm^3^)	243 ± 123	667 ± 276	<0.001
Lean Mass (kg)	46 ± 8	57 ± 9	<0.001
Age (yrs)	47 ± 10	42 ± 10	0.09
Height (m)	1.68 ± 0.06	1.67 ± 0.08	0.58

**Table 2:** Fasting blood results for the study population

	Normal Weight Subjects N = 16	Obese Subjects N = 38	p value
Fasting Blood Glucose (mmol/l)	5.0 ± 0.42	5.2 ± 0.6	0.223
Fasting Total Cholesterol (mmol/l)	5.1 ± 0.8	5.0 ± 0.8	0.66
Fasting HDL Cholesterol (mmol/l)	1.7 ± 0.4	1.3 ± 0.3	<0.01
Fasting Insulin (μm/l)	3.5 ± 5.8	11.1 ± 10.0	<0.01
Fasting Leptin (ng/l)	13 ± 18	119 ± 71	<0.001
HOMA-IR	0.82 ± 1.4	2.66 ± 2.58	0.01
C-Reactive Protein (mg/l)	0.2 ± 0.8	1.9 ± 3.2	<0.01

### Left Ventricular and Aortic Characteristics

Left ventricular mass and end-diastolic volume were both larger in the obese group when compared to the normal weight group. End-systolic volume, stroke volume and cardiac output were also elevated in obesity. Left ventricular ejection fraction was similar in normal weight and obese subjects (Table 3). Aortic distensibility in the ascending aorta was similar in the normal and obese group (4.56 ± 2.0 vs 4.63 ± 2.0 mmHg^-1 ^× 10^-3^, p = 0.90)

**Table 3:** Left Ventricular Characteristics for the Study Population

	Obese Subjects N = 38	Normal Weight Subjects N = 16	p value
Left Ventricular Mass (g)	126 ± 27	90 ± 20	<0.001
Left Ventricular End-diastolic Volume (ml)	146 ± 20	116 ± 22	<0.001
Left Ventricular End-systolic Volume (ml)	45 ± 11	37 ± 11	<0.001
Left Ventricular Stroke Volume (ml)	101 ± 15	79 ± 16	<0.001
Left Ventricular Ejection Fraction (%)	69 ± 5	68 ± 6	0.66
Cardiac Output (ml)	6.6 ± 1.3	4.8 ± 1.3	<0.001

### Relationship between Serum Markers of Obesity and Left Ventricular Mass

The associations of individual serum markers of obesity with left ventricular mass was determined using simple linear regression, with left ventricular mass as the dependent variable. Both leptin and insulin had a significant positive relationship with left ventricular mass. C-reactive protein and fasting serum glucose level were not related to left ventricular mass.

### Relationship between Anthropometric Data and Left Ventricular Mass

On simple linear regression, total fat mass, visceral fat mass, lean mass, waist hip ratio and BMI all showed a significant positive relationship with left ventricular mass. In addition to this both height and age had positive relationships with left ventricular mass. In this normotensive cohort, neither systolic nor diastolic blood pressure were related to left ventricular mass (Table 4).

**Table 4:** Linear Regression Analysis for Left Ventricular Mass

	R^2^	β	F	p value
Body Mass Index (kg/m^2^)	.263	.51	18.5	<0.001
Age (yrs)	.112	.34	6.6	0.013
Height (m)	.196	.44	12.7	0.001
Systolic Blood Pressure (mmHg)	.050	.22	2.7	0.11
Diastolic Blood Pressure (mmHg)	.052	.23	2.8	.09
Waist Hip Ratio	.292	.54	21.4	<0.001
Total Fat Mass (kg)	.152	.39	9.3	0.004
Visceral Fat Mass (dm^3^)	.388	.62	33.0	<0.001
Lean Mass (kg)	.701	.84	121.8	<0.001
Fasting Blood Glucose (mmol/l)	.087	.29	4.7	0.04
Fasting Insulin (μm/l)	.174	.42	10.9	0.02
Fasting Leptin (ng/l)	.079	.28	4.4	0.04
C-Reactive Protein (mg/l)	.075	.27	3.6	0.07
Left Ventricular End-diastolic Volume (ml)	.509	.71	53.2	<0.001
Left Ventricular End-systolic Volume (ml)	.153	.39	9.4	0.003
Left Ventricular Stroke Volume (ml)	.531	.73	58.9	<0.001
Cardiac Output (ml)	.244	.49	16.7	<0.001
Ascending Aortic Distensibility (mmHg^-1^×10^-3^)	.012	.11	0.6	0.44
Lean Mass Indexed to Height (g/m)	.699	.84	120.6	<0.001

### Relationship between Left Ventricular Function, Aortic Function and Left Ventricular Mass

On simple linear regression, left ventricular end-diastolic volume, end-systolic volume, stroke volume and cardiac output were all associated with left ventricular mass. Neither left ventricular ejection fraction nor ascending aortic distensibility were related to left ventricular mass (Table 4)

### Independent Predictors of Left Ventricular Mass

**Figure 2:**
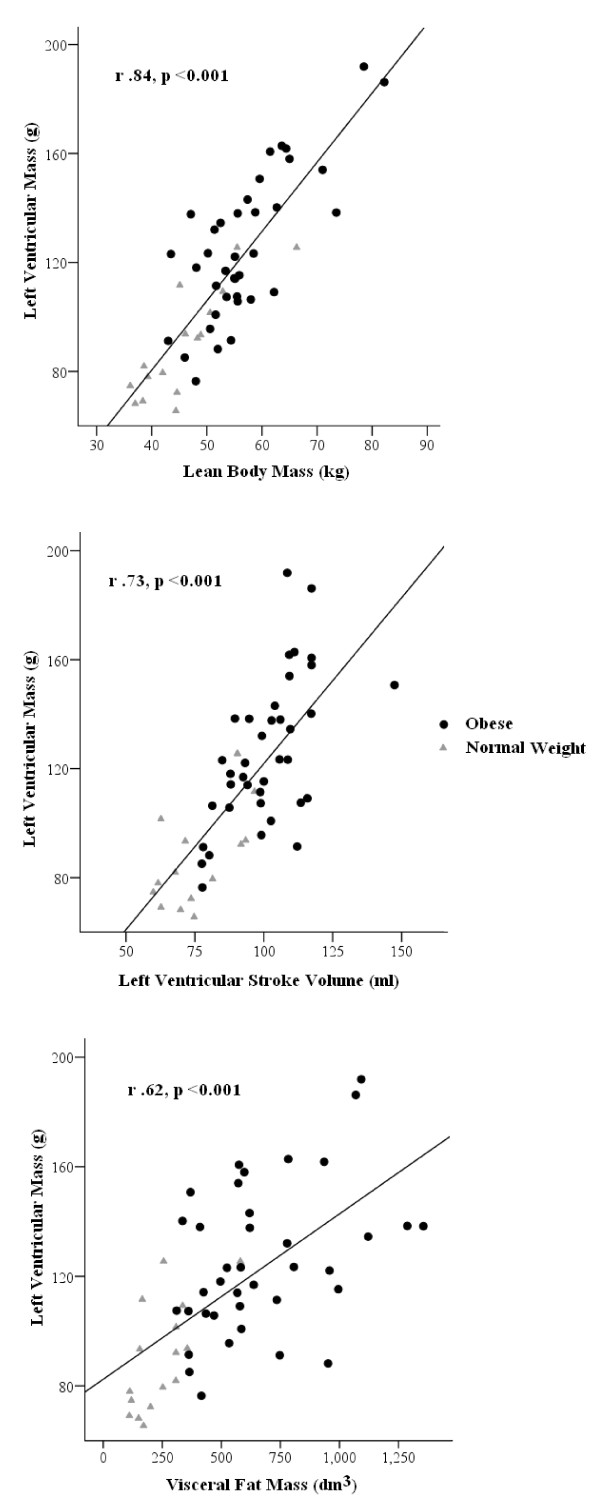
**Pearson Correlations for Independent Predictors of Left Ventricular Mass**.

To assess the major determinants of left ventricular mass, a stepwise multiple linear regression analysis of all data was performed. The multivariate model consisted of left ventricular mass as the dependent variable and of independent variables that had the most significant relationships with left ventricular mass in the simple linear regression analysis (visceral fat mass, total fat mass, lean body mass, height, age, insulin, and stroke volume, BMI). Multivariate analysis of left ventricular mass revealed lean body mass (β = 0.51, p < 0.001), left ventricular stroke volume (β = 0.31 p = 0.001) and visceral fat mass (β = 0.20, p = 0.02) as the main independent predictors of left ventricular mass (overall R^2 ^= 0.77, Figure 2).

## Discussion

In this study, we are the first group to use CMR to investigate the determinants of left ventricular mass in a severely obese population without additional co-morbidity and no other identifiable cardiovascular risk factors. In agreement with previous studies, we have again shown that obesity is associated with elevated left ventricular mass, cavity dilatation and increased cardiac output. [1] As a novel finding, we demonstrate that lean body mass, visceral fat mass and stroke volume are independent predictors of absolute left ventricular mass in this cohort.

The various determinants of left ventricular hypertrophy in the general population has been the subject of multiple research studies, but no study to date has investigated obesity without the confounders of obesity related comorbidites. Such studies have highlighted age, height, systemic arterial hypertension, and body size as the main predictors of left ventricular hyperrtophy. [8,28] In the setting of obesity accompanied by co-morbidities, insulin resistance, abdominal adiposity, systemic arterial hypertension, and lean mass have previously been shown to be the main predictors of left ventricular mass. [29-31] In order to dissociate the effects of obesity *per se *from the co-morbidities of obesity, which are known to exert independent effects on the myocardium, we now investigated obese subjects without hypertension or pathological insulin resistance.

### Lean Body Mass

The effect of increased lean body mass representing all non-adipose tissue, in determining left ventricular mass in an obese population with co-morbidities, has been established, with lean mass being more associated with left ventricular mass than total fat mass. [8,31] Here, we show, for the first time, that the major determinant of left ventricular mass in an obese population without additional cardiovascular risk factors and co-morbidity is also lean body mass. This could be explained by the fact increased body size, as a result of increased fat mass, would be expected to result in an associated increase in skeletal muscle mass in order to compensate for the additional physical demand of increased weight. As lean mass is responsible for the majority of the metabolic activity of the body and is one of the principle determinants of cardiac output, the strong association between increased left ventricular mass and increased lean mass is not surprising.

In addition to this, both left ventricular mass and lean body mass would also be expected to vary with intrinsic differences in body size i.e. height. In order to account for this variation in lean mass that occurs with intrinsic body size differences, which is not linked to increased cardiovascular risk, we investigated the association between lean body mass indexed to height and left ventricular mass. Interestingly, when lean mass was indexed to height the relationship with left ventricular mass remains as strong as that with absolute lean body mass (Table 4). This suggests not only that the increased lean mass seen here was not due to inherent differences in body size, but also that the left ventricular mass increases seen here are more likely related to increases in lean mass that occur secondary to obesity.

### Visceral Fat Mass

Visceral fat has been positively correlated with left ventricular mass in normotensive obese subjects and adipose tissue is one of main sources of the hormonal factors increasing left ventricular mass via non-haemodynamic mechanisms. [32]

Here again we show a strong positive correlation between visceral fat mass, as assessed by magnetic resonance imaging, and left ventricular mass. As the amount of intra-abdominal fat is now considered to be a risk factor for cardiovascular disease, establishing how visceral fat affects left ventricular characteristics is of increasing importance. Elevated visceral fat mass has been linked to subacute inflammation and hyperinsulinaemia, both of which have been previously shown to be correlated with left ventricular mass. [33] Hyperinsulinaemia, as a result of insulin resistance, is a potential explanation for the association between increased visceral fat mass and ventricular hypertrophy seen here. Hyperinsulinaemia has been linked to ventricular hypertrophy in obesity directly via the binding of insulin to myocardial insulin-like growth factor 1 receptors.[34] In this study, although insulin was seen to be a related to left ventricular mass on simple linear regression, it was not seen to be an independent predictor of left ventricular mass on multiple regression analysis. This points to the fact that visceral fat is acting through the composite of multiple mechanisms including hyperinsulinaemia, insulin resistance, inflammation and hyperleptinaemia, all of which in this study have smaller individual correlations with left ventricular mass.

### Stroke Volume

Left ventricular stroke volume has been shown to be a major determinant of left ventricular mass in various patient groups including hypertensive and overweight subjects [35,36]. In this study, we have shown that stroke volume is a strong predictor of left ventricular mass in obese individuals without associated hypertension. It is thought that the hypertrophic response of the left ventricle in obesity is, at least in part, secondary to increased wall stress imposed by cavity dilatation and stroke volume increases. [10,11] Here, we have shown that both end-diastolic volume and stroke volume are strongly associated with left ventricular mass on simple linear regression, and that stroke volume is an independent predictor of left ventricular mass in this group of patients. However, given the fact that in the setting of eccentric hypertrophy, the pattern seen here in obesity, increased end diastolic volume is inexorably linked to increased left ventricular mass it becomes unsurprising that as stroke volume is determined by end-diastolic volume, stroke volume itself is a major determinant of left ventricular mass.

### Hormonal and Inflammatory Changes

This study has confirmed the previous findings demonstrating positive correlations between serum marker of obesity i.e leptin and insulin and left ventricular mass changes. [12-15] However, although both hormones are well known to cause left ventricular hypertrophy, neither were independent predictors of left ventricular mass in a multiple regression model. This is most likely explained by the substantially greater effects of visceral fat mass, stroke volume and lean body mass on the left ventricle.

In this study on simple linear regression analysis there was again a statistically significant relationship between fasting serum glucose and left ventricular mass. This could be explained by the relationship between elevated fasting serum glucose and elevated serum insulin, which has both been linked to increased left ventricular mass and in this study was associated with increased left ventricular mass in this study. Interestingly, despite the documented link between elevated CRP and left ventricular mass, in this study there was no significant correlation between these two variables. However, a recent study by Mehta et al delineated that although LVH was associated with an inflammatory state as reflected in elevated CRP levels [37], this relationship appears to be mediated by comorbid conditions including hypertension, which was absent from our cohort.

### Limitations

The interpretation of multivariate analysis with multiple independent variables must be done so in the context of the relatively small nature of this study.

## Conclusion

Obesity is linked to increased mortality. The mechanisms behind this are poorly understood but may be related to elevated left ventricular mass, which is independently linked to mortality. As result of this, identification of the determinants of left ventricular mass in obesity, has implications for prognosis and therapeutic intervention aimed at reducing mortality. We have shown that lean body mass, visceral fat mass, and stroke volume all have a statistically significant independent association with left ventricular mass and together account for over 75% of the variance in this study. This suggests that all three factors play important roles in determining left ventricular mass in obesity uncomplicated by co-morbidities.

## Abbreviations

BMI: Body Mass Index; HOMA-IR: Homeostasis Model Assessment: Insulin Resistance; CMR: Cardiovascular Magnetic Resonance; SSFP: Steady State Free Procession; TE: Echo Time; TR: Repetition Time; SD: Standard Deviation.

## Competing interests

The authors declare that they have no competing interests.

## Authors' contributions

OR was involved in the design of the project, the data acquisition, analysis and drafting of the manuscript. JB was involved with patient recruitment, study design and statistical methods. JF and MA were involved in the data acquisition, and analysis SN, KC and SP conceived the study, and participated in its design and coordination and helped to draft the manuscript. All authors read and approved the final manuscript.
